# Supplementary figures and images for: CYRI controls epidermal wound closure and cohesion of invasive border cell cluster in Drosophila
Source: J Cell Biol. 2024 Oct 25;223(12):e202310153. doi: 10.1083/jcb.202310153 (PMC11519390; doi:10.1083/jcb.202310153)

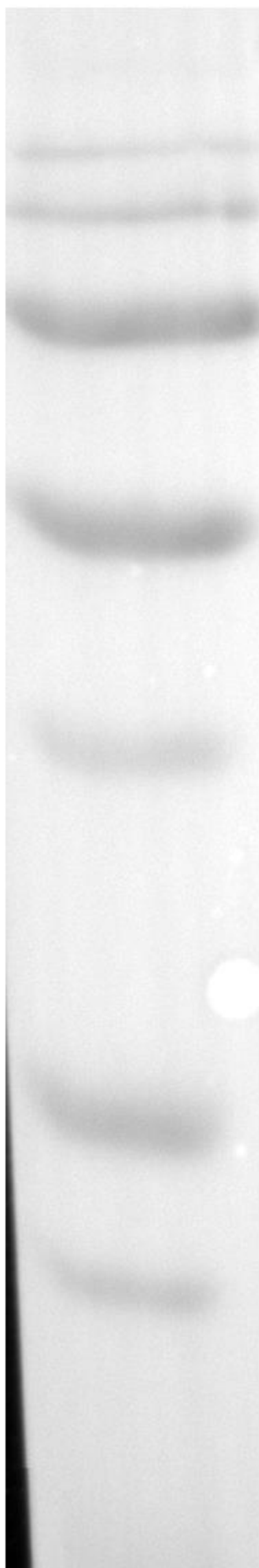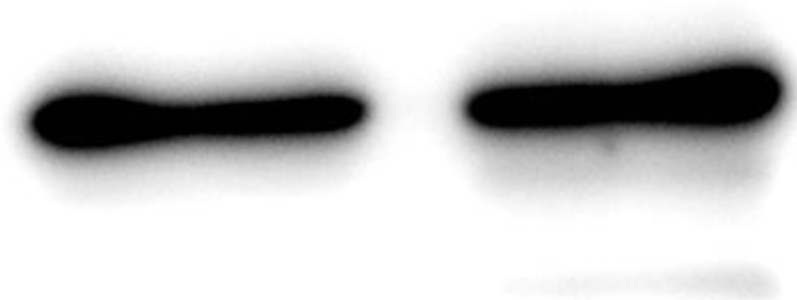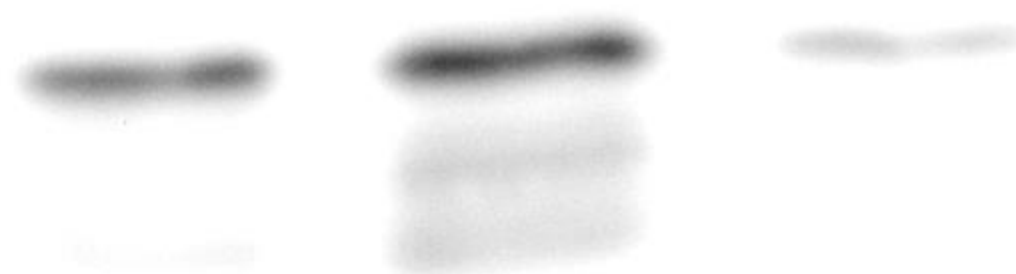

Supplement: SourceData F1 — is the source file for Fig. 1. [file JCB_202310153_SourceDataF1.pdf]

anti-Cyri

1

2

3

4

75

50

37

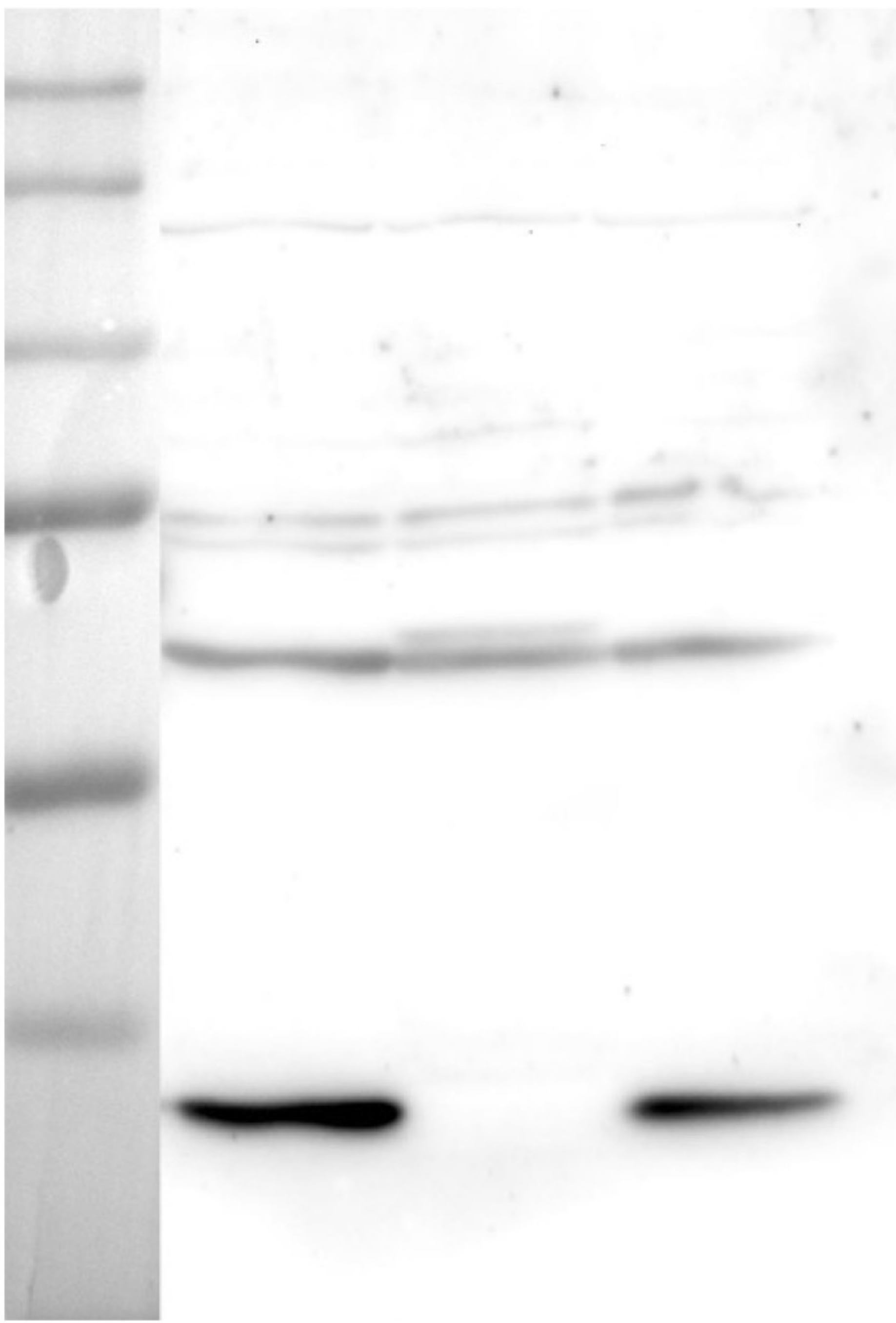

1

2

3

4

75

50

37

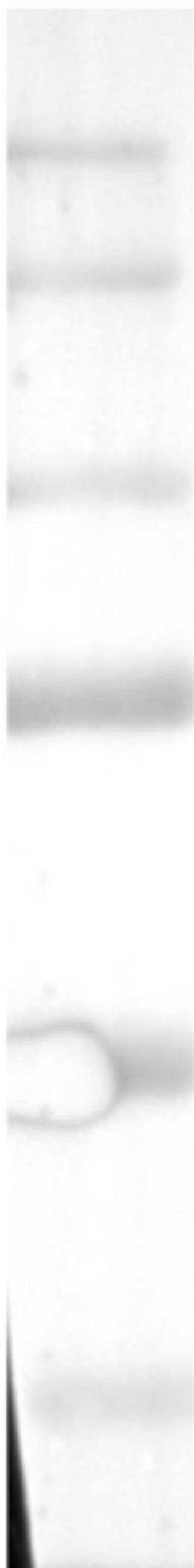

Supplement: SourceData F2 — is the source file for Fig. 2. [file JCB_202310153_SourceDataF2.pdf]

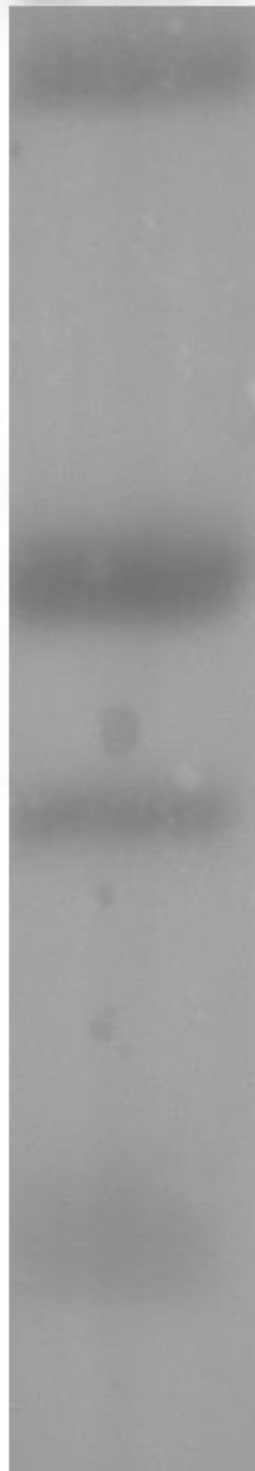

1

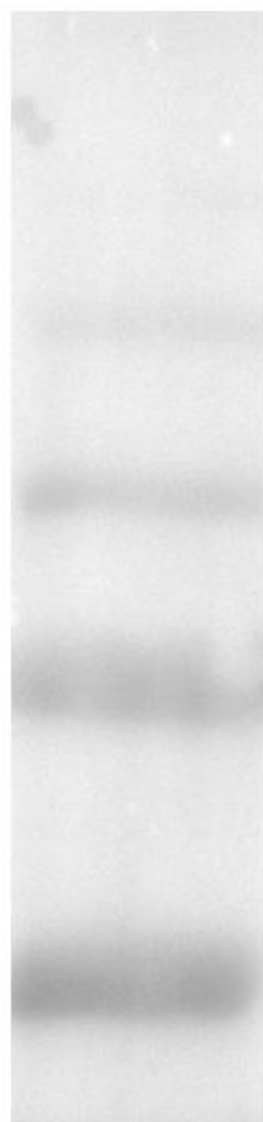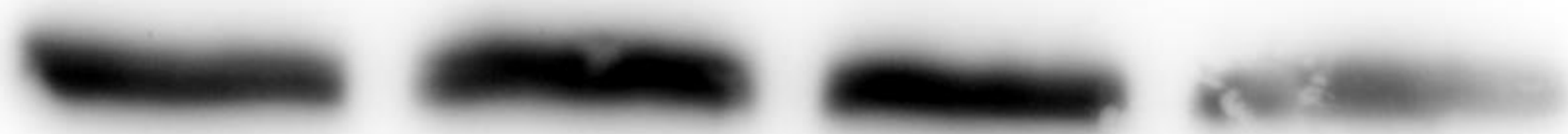

Supplement: SourceData F3 — is the source file for Fig. 3. [file JCB_202310153_SourceDataF3.pdf]

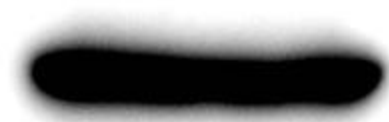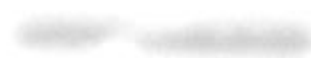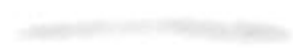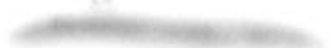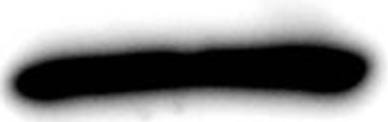

Supplement: SourceData FS1 — is the source file for Fig. S1. [file JCB_202310153_SourceDataFS1.pdf]

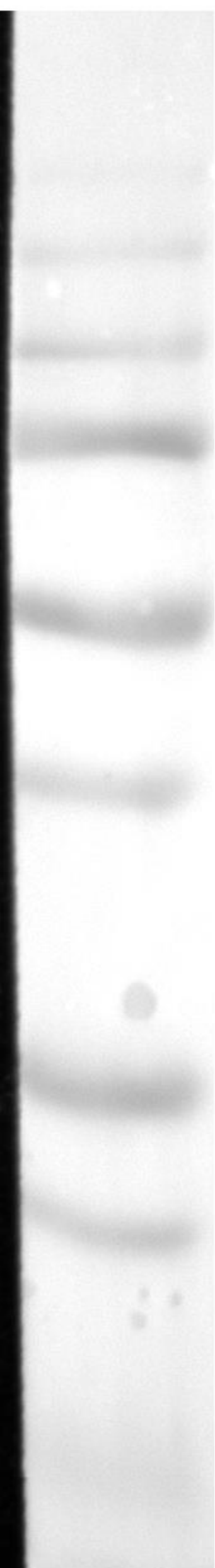

1

1

1

1

1

1

Supplement: SourceData FS2 — is the source file for Fig. S2. [file JCB_202310153_SourceDataFS2.pdf]

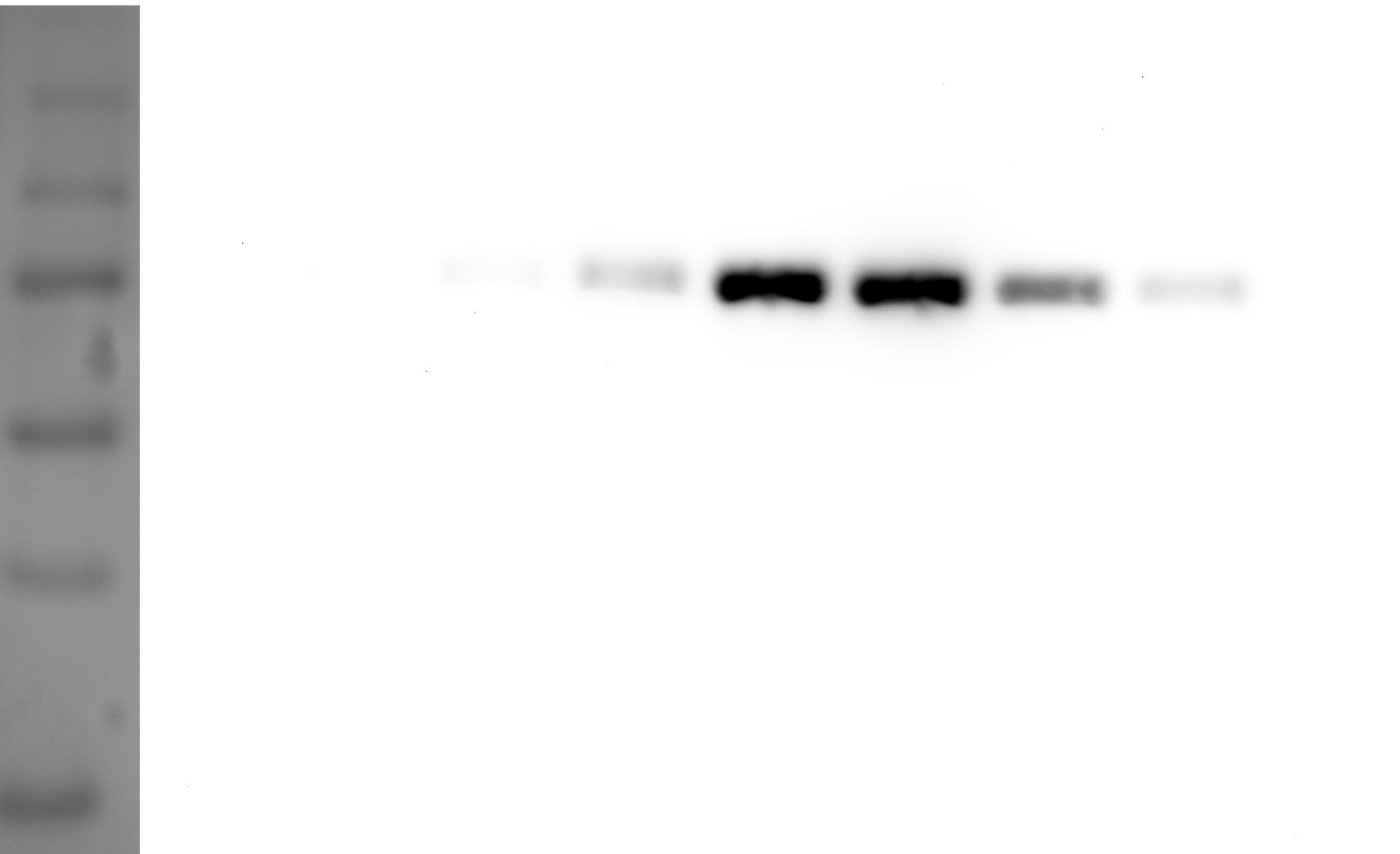

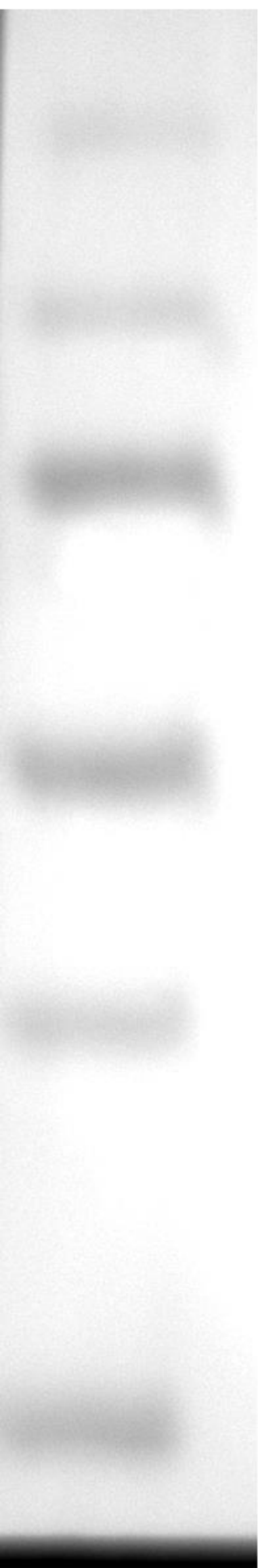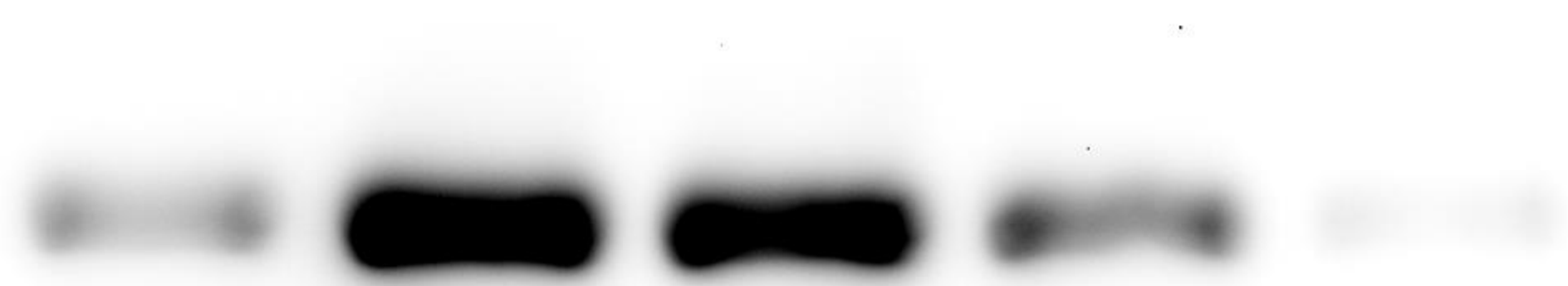

Supplement: SourceData FS4 — is the source file for Fig. S4. [file JCB_202310153_SourceDataFS4.pdf]
